# Long-Term Cognitive Aging Trajectories as Predictors of Daily Affect

**DOI:** 10.1093/geroni/igab046.1282

**Published:** 2021-12-17

**Authors:** Denis Gerstorf, Oliver Schilling, Ute Kunzmann, Martin Katzorreck, Anna Lücke, Hans-Werner Wahl, Nilam Ram

**Affiliations:** 1 Humboldt University of Berlin, Humboldt University of Berlin, Brandenburg, Germany; 2 University of Heidelberg, Heidelberg, Baden-Wurttemberg, Germany; 3 Leipzig University, Leipzig, Sachsen, Germany; 4 University of Leipzig, Leipzig, Sachsen, Germany; 5 Heidelberg University, Heidelberg, Baden-Wurttemberg, Germany; 6 Stanford University, Stanford, California, United States

## Abstract

Multiple-time scale studies provide new opportunities to examine how developmental processes evolving on different cadences are intertwined. Theories about age-related accumulation of stress suggest that long-term progressive loss of cognitive resources should manifest in and shape short-term daily affective experiences. Applying growth modeling and intraindividual variability methods to data obtained from 123 young-old (65-69 years, 51% women) and 47 very-old adults (85-88 years, 49% women) who provided 20+ year longitudinal data on Digit Symbol performance and 42-occasion momentary data about the emotions and stressors they experienced during everyday life (6 reports per day), we found that shallower long-term loss of cognitive performance was associated with less fluctuation in momentary positive affect, as well as less “spikiness” of and reactivity to stress. We discuss and present further results highlighting how mid-term processes surrounding age, gender roles, and health additionally contribute to and shape links between long-term and short-term dynamics of aging.

